# Book Notices

**Published:** 1868-06-01

**Authors:** 


### *BOOK NOTICES.*

---

Researches in Obstetrics. By J. Matthews Duncan, A.M., M.D., L. R. C. S. E., Lecturer in Surgeon's Hall [London] Medical School, etc., etc. New York: Wm. Wood & Co., 71 Walker street. 1868. Pp. 467. Wm. B. Keen & Co., 148 Lake street, Chicago.

This work is brought out in very superior style by the eminent publishing house whose imprint appears on the title page. The contents are mainly collected from the author's writings in various medical and scientific periodicals — the whole carefully revised or rewritten. A few are now for the first time in print. The student and practitioner will find in it a large amount of valuable information, and suggestions which can not be obtained in the usual systematic treatises on the subject.

Felix Von Niemeyer's Clinical Lectures on Pulmonary Phthisis. Translated, by permission of the Author, from the Second German Edition, by J. L. Parke. New York: Moorhead, Simpson & Bond. 1868. Pp. 116. From the Publisher.

A very valuable monograph, which, although we dissent from many of the views of the author, we commend to the perusal of our readers.

The Indigestions; or Diseases of the Digestive Organs Functionally Treated. By Thomas King Chambers, Honorary Physician — [But what is the necessity of saying or writing who Chambers is?] Second American from the Second and Revised English Edition. Philadelphia: Henry C. Lea. 1868. Chicago: S. C. Griggs & Co.

The Journal thinks no living writer on Practice of Medicine has done more for its real advancement than Thomas King Chambers. He has devoted attention to the "roots of the tree of life," and finds that they are best supplied by the elements of nutrition. Mere medication has very little to do with it. *O si sic omnes!* All medication at the present savors of empiricism, which does not refer constantly to nutrition. All physicians who wish to keep pace with the times should carefully peruse the writings of this eminent author.

On Diseases of the Skin; A System of Cutaneous Medicine. By Erasmus Wilson, F. R. S. Seventh American from the Sixth and Revised English Edition. With Twenty Plates. and Illustrations on Wood. Philadelphia: Henry C. Lea. 1848. Pp. 808. Chicago: S. C. Griggs & Co.

It is with pleasure we chronicle a new edition of this, the best treatise on diseases of the skin extant.

